# A unified multi-kingdom Golden Gate cloning platform

**DOI:** 10.1038/s41598-019-46171-2

**Published:** 2019-07-12

**Authors:** David Chiasson, Victor Giménez-Oya, Martin Bircheneder, Sabine Bachmaier, Tanja Studtrucker, Joel Ryan, Katharina Sollweck, Heinrich Leonhardt, Michael Boshart, Petra Dietrich, Martin Parniske

**Affiliations:** 1 Faculty of Biology, Genetics, LMU Munich, D-82152 Martinsried, Germany; 2 Faculty of Biology and Center for Integrated Protein Science Munich (CIPSM), LMU Munich, Großhaderner Str. 2, D-82152 Martinsried, Germany; 3 Department Biology, Friedrich-Alexander-Universität Erlangen-Nürnberg, D-91058 Erlangen, Germany; 4 Present Address: Department of Biology, Saint Mary’s University, B3H 3C3 Halifax, Canada

**Keywords:** Molecular biology, Expression systems, Genetic techniques, Genetics

## Abstract

Assembling composite DNA modules from custom DNA parts has become routine due to recent technological breakthroughs such as Golden Gate modular cloning. Using Golden Gate, one can efficiently assemble custom transcription units and piece units together to generate higher-order assemblies. Although Golden Gate cloning systems have been developed to assemble DNA plasmids required for experimental work in model species, they are not typically applicable to organisms from other kingdoms. Consequently, a typical molecular biology laboratory working across kingdoms must use multiple cloning strategies to assemble DNA constructs for experimental assays. To simplify the DNA assembly process, we developed a multi-kingdom (MK) Golden Gate assembly platform for experimental work in species from the kingdoms Fungi, Eubacteria, Protista, Plantae, and Animalia. Plasmid backbone and part overhangs are consistent across the platform, saving both time and resources in the laboratory. We demonstrate the functionality of the system by performing a variety of experiments across kingdoms including genome editing, fluorescence microscopy, and protein interaction assays. The versatile MK system therefore streamlines the assembly of modular DNA constructs for biological assays across a range of model organisms.

## Introduction

The generation of the first chimeric DNA molecule containing bacteriophage lambda DNA inserted into the SV40 viral genome was a key event in the discipline of molecular genetics^1^. Subsequently, researchers created the first recombinant bacterial DNA molecule containing eukaryotic DNA^2^. The generation of such chimeras has since become standard practice in molecular biology laboratories and is a fundamental requirement for the emerging discipline of synthetic biology. Synthetic biology aims to redesign existing biological systems and generate novel biological organisms with synthetic parts. Achieving these goals requires the assembly of complex genetic material. To build such genetic assemblies a number of cloning techniques have been developed. Seamless methods such as Gibson assembly^3^, USER cloning^4^, and In-fusion^5^ have been used successfully to stitch together pieces of DNA. However, these methods require amplification of fragments with custom overlapping sequences and are not modular by nature. In contrast, Golden Gate^6^ is a modular cloning system which facilitates constructing custom DNA molecules.

Golden Gate cloning utilizes type IIS (shifted cleavage) restriction enzymes to accurately assemble DNA parts in a defined order. Type IIS enzymes recognize non-palindromic DNA sequences and cut the DNA backbone outside of the recognition site. In contrast, typical type IIP (palindromic) restriction enzymes recognize a palindromic sequence and generally cut the DNA within the recognition site. Since the cut site of type IIS enzymes is not contained within the recognition sequence, the staggered overhangs produced after cleavage can be custom designed for modular cloning. The enzymes BsaI (Eco31I), BpiI (BbsI), and Esp3I (BsmBI) are commonly used to generate custom 4 base pair (bp) overhangs, while rare cutters SapI (3 bp overhang) and AarI (4 bp overhang) have recently been utilized for modular cloning^7–9^. Binder *et al*.^10^ developed a Golden Gate modular system for plants based upon BsaI, BpiI, and Esp3I enzymes for assembling custom DNA parts. The 4 bp overhang sequences were chosen such that the sequence of bases in positions #2 and #3 was only used once to ensure high fidelity assembly. We had observed that enzyme preparations often contain contaminating exonucleolytic activity, resulting in the cleavage of the 5′ base in position #1. The removal of the 5′ base can lead to annealing and ligation of overhangs that are not a perfect match. Restricting the sequence of bases 2 and 3 to a unique dinucleotide therefore limits the number of possible 4 bp overhangs to 16. A recent high-throughput analysis of end-joining ligation fidelity has identified additional Golden Gate junction sequences for precise assembly^11^.

Since the first description of a Golden Gate cloning system^6^, numerous modular systems have emerged for working with model organisms. The original Modular Cloning (MoClo) system was developed for working with plants^12^ and has since been expanded for additional applications^13–15^. The GoldenBraid^16–18^ and GreenGate^19^ and Binder *et al*.^10^ modular systems were also developed for plant research. Although the plant Golden Gate cloning systems rely upon the similar type IIs enzymes, they may differ in overhangs, plasmid backbones, and cloning workflow. Since parts from each system are modified to remove type IIs restriction enzyme sites, they can be reamplified by PCR with compatible overhangs for another platform. Following the work in plants, Golden Gate cloning systems have since been developed for bacteria^7,20–22^, yeast^23–26^, and human cells^27,28^. Although each system works well for the intended organism, a typical molecular biology laboratory utilizes multiple organisms to assess protein function. Thus, there is a need for a universal Golden Gate cloning system to facilitate assembling DNA constructs for cross-kingdom experimental work.

## Results

Since existing Golden Gate systems are generally only applicable to target organisms from one kingdom, we sought to develop a universal system so that constructs required for experiments in typical model organisms could be generated with the same procedures. Given that DNA parts originating from organisms with variable codon usage frequencies can generally be used in heterologous hosts, we created a multi-kingdom (MK) modular cloning system. Our aim was to streamline the procedure for generating composite DNA molecules intended for use in gram-negative bacteria (Eubacteria), yeast (Fungi), plants (Plantae), trypanosomes (Protista), along with human cells and frog oocytes (Animalia). With such a platform, DNA elements (e.g. promoters, fusion tags, genes) can be interchanged across multiple kingdoms using a single cloning strategy, streamlining plasmid construction in the laboratory.

### General cloning strategy to enter the MK system

**Figure 1:**
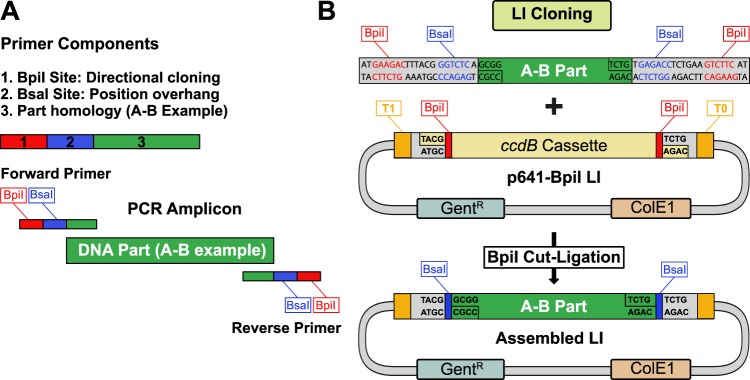
MK system entry cloning strategy. (**A**) Overview of required primer components for amplifying a new DNA part (A-B example). In addition to part homology, each primer contains a BpiI restriction site for directional LI cloning, and a BsaI restriction site for LII cloning. (**B**) Performing a BpiI cut-ligation with the amplified part and the universal p641-BpiI backbone generates a LI entry clone for the MK system. The p641-Bpi plasmid contains T1 and T0 terminator sequences flanking the cloned insert, a gentamicin resistance cassette, and *ccdB* negative selection.

The MK cloning system is built upon the architecture of an existing Golden Gate cloning system originally designed for plants^10^. To enter the MK system, a fragment is first amplified by PCR with primers (Supplementary Fig. S1) containing BpiI restriction sites for directional cloning into the entry vector and BsaI restriction sites to define the part position (Fig. 1A). By default, DNA parts are cleared of BsaI, BpiI, and ideally Esp3I restriction sites. Incompatible restriction enzyme sites can be removed during entry cloning by introducing silent mutations with overlapping primers^10^. New parts are then cloned into the p641-BpiI acceptor backbone (Fig. 1B) using a “cut-ligation” protocol (Supplementary Fig. S2). The p641-BpiI backbone is based upon pSEVA641^29^ and carries a *ccdB* negative selection cassette along with T1 and T0 flanking terminator sequences to prevent read-through transcription. In case it is not desirable to remove BpiI restriction sites from a new part (e.g. a promoter), an alternative acceptor plasmid (p641-Esp3I) was generated for Esp3I-based cloning (Supplementary Fig. S3). Parts can also be cloned without any mutagenesis by either TA cloning (p191-TA) or blunt cloning into pUC57-based plasmids using blue-white screening (Supplementary Fig. S3).

### Building transcription units and higher order DNA assemblies

**Figure 2:**
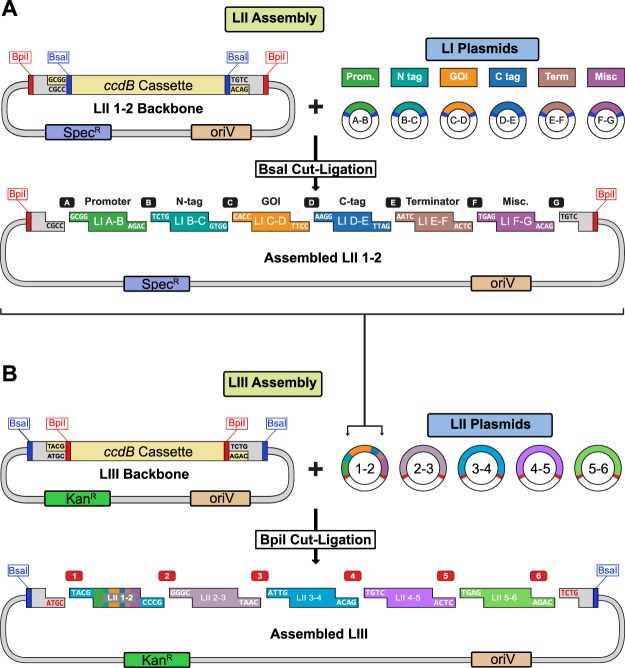
Creating LII and LIII plasmids with the MK cloning system. (**A**) LI plasmids containing DNA parts (A-B, B-C, C-D, D-E, E-F, F-G positions) are mixed with a LII backbone in a BsaI cut-ligation to generate a LII plasmid. LII backbones contain a spectinomycin resistance cassette and a *ccdB* negative selection cassette. (**B**) An assembled LIII plasmid is created by mixing LII plasmids (1-2, 2-3, 3-4, 4-5, 5-6 positions) with a LIII backbone in a BpiI cut-ligation. LIII backbones contain kanamycin resistance and *ccdB* negative selection cassettes.

**Figure 3:**
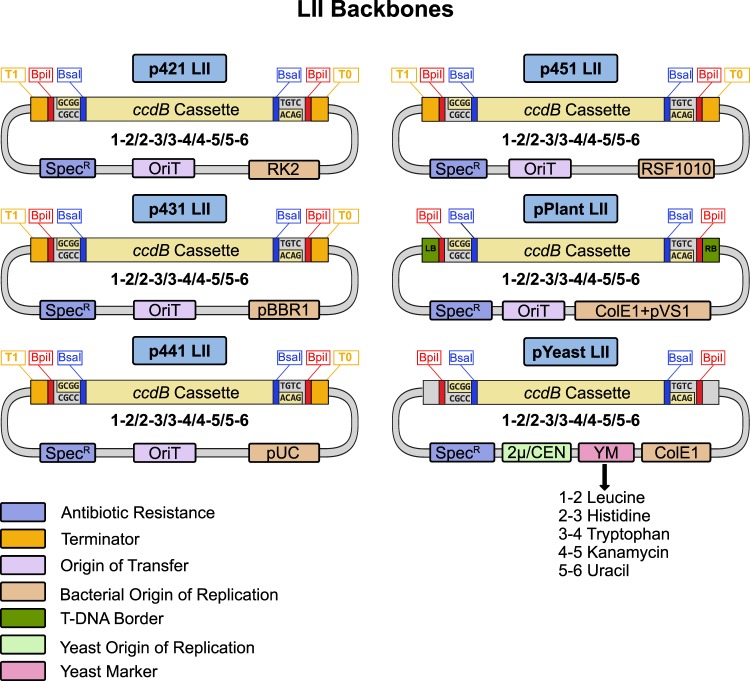
Overview of the LII MK backbones. The system contains six unique LII plasmid backbone series (each with positions 1-2, 2-3, 3-4, 4-5, 5-6). The p421 (RK2), p431 (pBBR1), p441 (ColE1), and p451 (RSF1010) series contain different origins of replication for gram-negative bacteria, an origin of transfer (OriT), and flanking terminators (T1, T0). Plant backbones contain a double origin of replication for *E. coli* (ColE1) and *Agrobacterium* (pVS1) along with an OriT and T-DNA borders for genomic integration. Yeast backbones contain a ColE1 origin of replication for bacteria, a yeast origin of replication (high copy 2 µ or low copy CEN/ARS), and an auxotrophic or dominant marker.

LI DNA parts are flanked by BsaI sites which generate unique overhangs to define the position (A-B, B-C, C-D, D-E, E-F, F-G) for LII assembly. Generally, promoters are cloned as A-B, N-terminal protein fusions as B-C, genes or open-reading frames as C-D, C-terminal protein fusions as D-E, terminators as E-F, and additional parts as F-G (Fig. 2A). LI dummies (DMY) are available to fill in any positions that are not required^10^. LI parts are combined into a transcriptional unit by BsaI cut-ligation into LII backbones. Negative *ccdB* selection along with a different antibiotic resistance (spectinomycin) ensure that only assembled LIIs are obtained. In order to expand the collection, we generated five new LII backbone plasmid series (Fig. 3). LII backbones with a range of bacterial origins of replication (RK2, pBBR1, ColE1, and RSF1010) were created to facilitate experiments in diverse organisms. New series of yeast backbones were also constructed with either high (2 µ) or low copy (CEN/ARS) origins of replication and auxotrophic (leucine, histidine, tryptophan, uracil) or dominant (kanamycin) markers for selection. All LII backbone series contain five plasmids with flanking BpiI sites to generate unique overhangs to define the position (1-2, 2-3, 3-4, 4-5, 5-6) for LIII assembly. Assembled LIIs in any backbone can then be combined by BpiI cut-ligation to generate LIII plasmids with up to five transcription units (Fig. 2B). LIII backbones with kanamycin selection for each series were also created for building higher order assemblies (Supplementary Fig. S4).

DNA parts are normally cleared of BsaI, BpiI, and ideally Esp3I restriction sites before entering the system. In some cases, it is not desirable to remove restriction sites by mutagenesis to avoid altering the original DNA sequence (e.g. a cis-regulatory element in a promoter part). Additionally, a DNA part may contain numerous type IIs restriction sites, making mutagenesis challenging. To circumvent mutagenesis, the MK system contains either *lacZ* or *ccdB* DMY landing pad cassettes (A-B or C-D position) for cloning into LII or LIII plasmids with alternative strategies. Supplementary Fig. S5 outlines the use of the Esp3I-lacZ-Esp3I and BsaI-*ccdB*-BsaI A-B DMYs (same procedures apply for C-D DMYs). The LI Esp3I-lacZ-Esp3I A-B is initially added to a LII assembly by BsaI cut-ligation and can be further incorporated into a LIII using the standard workflow. The lacZ cassette contains internal Esp3I sites with overhangs compatible for subsequently inserting an A-B part by Esp3I cut-ligation. Therefore, A-B (or C-D) parts containing BsaI or BpiI sites can be directly cloned into a LII or LIII without mutagenesis. The BsaI-*ccdB*-BsaI A-B (or C-D) cassette (Supplementary Fig. S5) can also replace the Esp3I-lacZ-Esp3I DMY in a LIII plasmid by Esp3I cut-ligation. The *ccdB* cassette enables insertion of LI parts containing either BpiI or Esp3I sensitive sites directly into a LIII by BsaI cut-ligation. Therefore, the developed cassettes enable cloning and integration of DNA parts into the MK system without modifying the original DNA sequence.

### Compatibility with Gateway cloning

Given that many large Gateway plasmid collections are available, we constructed a compatible entry backbone and parts for making destination backbones via Golden Gate cloning (Supplementary Fig. S6). The pENTR-BsaI-Tet backbone was created for sub-cloning of LI C-D parts by BsaI cut-ligation to generate Gateway entry clones. The pENTR-BsaI-Tet entry clone can be then be transferred to an existing Gateway destination plasmid by standard recombination with LR Clonase. The original kanamycin cassette of pENTR was exchanged for a tetracycline resistance cassette to facilitate selection of destination clones which are kanamycin resistant. LI Gateway parts with flanking recombination sites (*attR1-ccdB-Tet-attR2*) were also generated for positions A-B and C-D (Supplementary Fig. S6). The chloramphenicol resistance marker in the *ccdB* cassette was replaced by a tetracycline marker to enable selection of correctly assembled destination backbones in bacteria. Custom Gateway destination backbones can therefore be created in combination with Golden Gate assembly. As a result, the MK system can be readily used with existing Gateway plasmid collections.

### Protein localization and transport assays in animal cells

**Figure 4:**
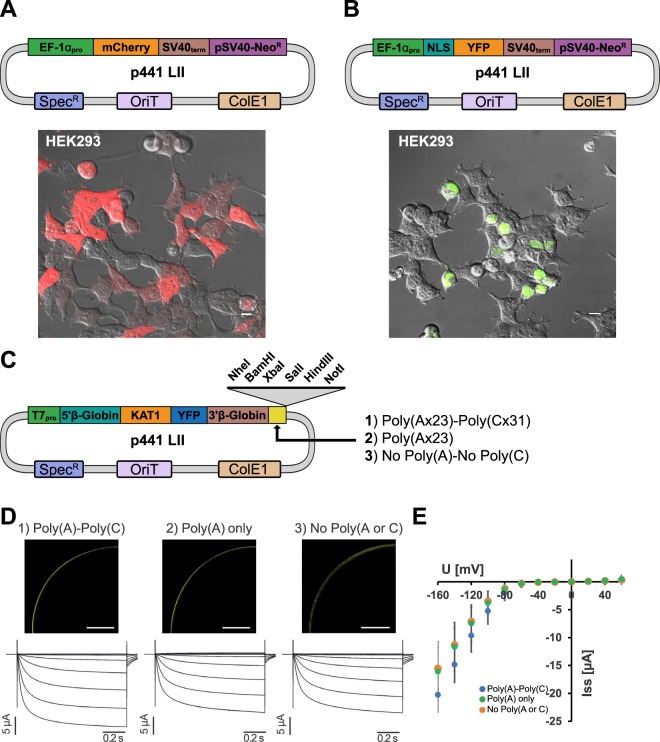
Characterization of MK components in human cells and *Xenopus* oocytes. Image overlays (fluorescence and DIC) of HEK293 cells transfected with LII reporter plasmids expressing mCherry (**A**) and NLS-YFP (**B**) driven by the ELF-1α promoter. (**C**) Construct design for functional expression of the KAT1 K^+^ channel in *Xenopus laevis* oocytes. Three independent DNA sequences placed downstream of the β-Globin 3′UTR were tested in parallel to monitor their effect on channel expression. Unique enzyme sites for plasmid linearization prior to cRNA synthesis are shown. (**D**) *Upper panel:* Confocal fluorescence images of oocytes injected with *KAT1-YFP* cRNA followed by the 3′ sequences shown in (**B**) (scale bars: 200 μm). L*ower panel*: Current voltage curves and representative current traces of oocytes micro-injected with *KAT-YFP* cRNA as indicated. (**E**) Steady state currents for the corresponding curves in (**D**). Shown are the measurement means and standard deviations. Voltage steps ranged from +60 mV to −160 mV in 20 mV decrements. Holding potential was set to −40 mV. Scale bars in (**A**) represent 10 µm and 200 µm in (**D**).

To demonstrate the functionality of the MK system, we performed a range of experiments in diverse organisms. Human embryonic kidney (HEK) cells were selected for protein localization assays. To enable expression, the elongation factor EF-1α promoter (A-B), SV40 terminator (E-F), and a neomycin cassette (F-G) were cloned as LIs. These components were combined into a LII plasmid (p441 backbone) with either untagged mCherry or NLS-YFP parts (Fig. 4A,B). The assembled LII plasmids were then transfected into HEK cells for imaging. mCherry was visible throughout the cell while NLS-YFP was detected in nuclei as anticipated. The p441 backbone was also used to assemble plasmids containing the *Arabidopsis thaliana* guard cell K^+^ channel KAT1 fused to YFP for expression in *Xenopus laevis*. We generated an A-B part containing a T7 promoter combined with the *X. laevis* β-globin 5′UTR and an E-F part containing the β-globin 3′UTR and poly(A) sequences from the commonly used plasmid pGEMHE^30^. The p441 plasmid backbone also contains 6 unique 3′ restriction enzyme sites for linearization prior to cRNA synthesis. Notably, the original pGEMHE plasmid contains a stretch of 31 cytosine bases (poly(C)) 3′ of the β-globin poly(A) sequence which were acquired during cloning process from a cDNA library^31^. We therefore created two additional E-F variants where either the poly(C) or the poly(C) and poly(A) sequences were deleted (Fig. 4C) to test the effect of these sequences on channel expression. Similar expression of KAT1-YFP channels was observed by confocal microscopy for all three constructs (Fig. 4D). The induction of typical voltage-dependent, slowly activating inward rectifying K^+^ currents upon hyperpolarization was also comparable. The current-voltage curves in Fig. 4E further show that the voltage-dependence and current densities were similar irrespective of the sequence following the 3′UTR. These data show that the assembled constructs are readily expressed in different organisms, using different delivery methods.

**Figure 5:**
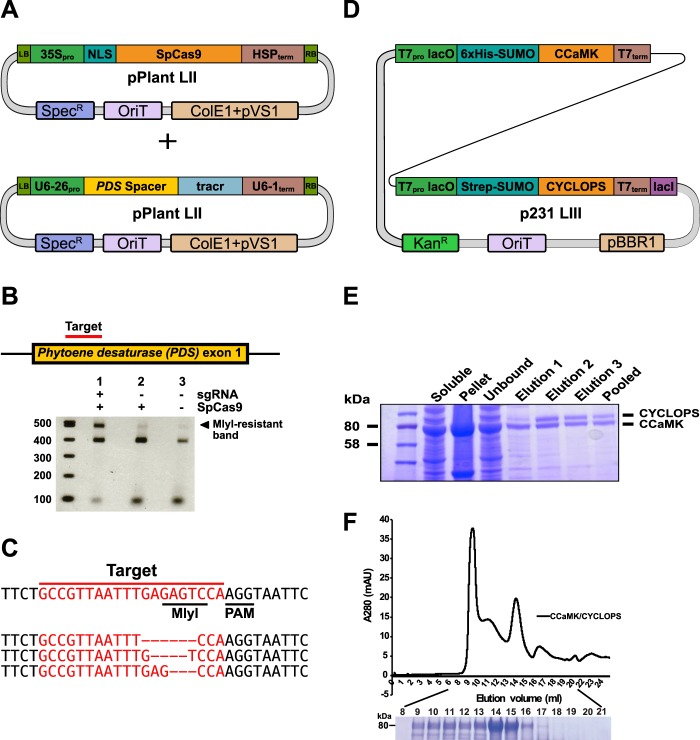
Genome editing in plants and recombinant protein purification. (**A**) Overview of LII plasmids used for genome editing of the *PDS* locus in *Nicotiana benthamiana* with SpCas9. (**B**) Illustration of exon 1 of the *PDS* locus with the target region (top) and an agarose gel showing the amplified *PDS* region after incubation with the MlyI restriction enzyme (below). (**C**) Sequencing of MlyI-resistant band clones uncovered indels in the target region. (**D**) Overview of the LIII plasmid containing *StrepII-SUMO-CYCLOPS* and *His-SUMO-CCaMK* for co-expression. (**E**) SDS-PAGE analysis of StrepII-SUMO-CYCLOPS and His-SUMO-CCaMK proteins after co-expression and purification by StrepII affinity chromatography. (**F**) The CCaMK-CYCLOPS complex was resolved by size exclusion chromatography. Shown below is an SDS-PAGE gel with lanes numbered according to elution volume. Full-length gels from (**E**,**F**) are presented in Supplemental Fig. 8.

### Genome editing *in planta*

For genome editing experiments in plants, the *phytoene desaturase* (*PDS*) locus of *Nicotiana benthamiana* was chosen as a target^32^. The target site contains an MlyI restriction site that is predicted to be disrupted upon mutagenesis. A plant codon-optimized *SpCas9*^33^ driven by the constitutive 35S promoter was assembled into a LII pPlant plasmid for integration into the plant genome (Fig. 5A). A second LII plasmid containing the *U6-26* promoter from *Arabidopsis thaliana* fused to a *PDS* gRNA followed by the *U6-1* terminator was also generated (Supplementary Fig. S7). The two plasmids were transferred into *Agrobacterium tumefaciens* and co-infiltrated into *N. benthamiana* leaves. After 2 days, leaf tissue was collected and the target *PDS* region was amplified by PCR and digested with MlyI. A digestion-resistant band was isolated, cloned, and sequenced (Fig. 5B). We observed a variety of indel mutations in the *PDS* gene (Fig. 5C) indicating that the locus was successfully targeted by SpCas9. Thus, the MK system can be used to easily assemble and express gRNA and Cas9 components for genome engineering.

### Recombinant protein expression in bacteria and interaction assays in yeast

**Figure 6:**
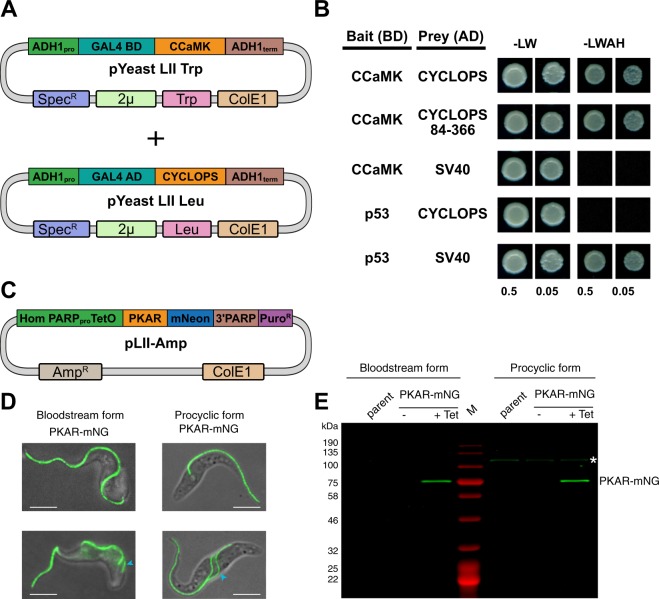
Protein interaction assay in yeast and protein localization in trypanosomes. (**A**) Overview of the LII plasmids used for yeast-two hybrid (Y2H) experiments. (**B**) Y2H interaction assay between CCaMK fused to the GAL4 binding domain (BD) and CYCLOPS fused to the GAL4 activation domain (AD). A deletion variant of CYCLOPS (amino acids 84–366) delineates the interaction domain. p53 (amino acids 72–390) and SV40T (amino acids 87–708) were cloned and used as interaction controls. Yeast cells were resuspended in water (OD_600_ = 0.5 and 0.05) and spotted onto −LW (leucine, tryptophan) and −LWAH (leucine, tryptophan, adenine, histidine) solid media. (**C**) LII construct used to integrate a tetracycline (Tet)-inducible fusion of the protein kinase A regulatory subunit (PKAR) to mNeonGreen (mNG) into the genome of *Trypanosoma brucei*. (**D**) Fluorescence microscopy of *T. brucei* cells expressing PKAR-mNG. Expression was induced by treatment with 1 µg/ml tetracycline for 24 h in two different life cycle stages of the parasite, long slender bloodstream forms (left) and procyclic forms (right). Fluorescence microscopy images show the localization of the PKAR-mNG fusion protein in Tet-induced cells. Trypanosomes with a single flagellum in G1 phase of the cell cycle are displayed in the upper images. Dividing cells having two flagella are shown on the lower images; the daughter flagellum is marked by a blue arrowhead. Scale bar 5 µM. (**E**) In-gel fluorescence of the *T. brucei* parental cell line, non-induced (−Tet) and Tet-induced (+Tet) cell extracts. The white asterisk (*) marks an endogenous, autofluorescent protein of unknown identity expressed in procyclic stage parasites.

A protein complex containing the *Lotus japonicus* calcium- and calmodulin-dependent protein kinase (CCaMK) and the transcriptional activator CYCLOPS was chosen for a co-expression and purification assay in *E. coli*. Upon binding calcium and calmodulin, CCaMK is activated and phosphorylates CYCLOPS^34^. Golden Gate parts were generated for T7-based expression in *E. coli*. Two level II plasmids (*T7:His-SUMO-CCaMK* and *T7:StrepII-SUMO-CYCLOPS*) were combined into the p231 LIII backbone for co-expression (Fig. 5D). Affinity purification of CYCLOPS was used to isolate the complex from the bacterial lysate. Two bands corresponding to CCaMK and CYCLOPS were identified by SDS-PAGE analysis of the eluted fractions indicating that both proteins were isolated from a complex (Fig. 5E). Subsequently, the pooled fractions were subjected to size-exclusion chromatography. A faster migrating (higher molecular weight) complex was eluted from the column which contained both CCaMK and CYCLOPS (Fig. 5F). The interaction of CCaMK and CYCLOPS was also analyzed using a yeast two-hybrid (Y2H) assay. The Gal4 binding domain (Gal4BD, B-C part) was fused to CCaMK and the Gal4 activation domain (Gal4AD, B-C part) fused to CYCLOPS (Fig. 6A). The murine p53 and simian virus 40 (SV40) T antigen were cloned as controls. Expression of the Gal4 fusions was driven by variants of the *alcohol dehydrogenase 1* (*ADH1)* promoter in either the pYeast LII Leu 1-2 or pYeast LII Trp 3-4 backbones. Co-transformation of CCaMK and CYCLOPS resulted in growth on selection (−LWAH) plates indicating an interaction (Fig. 6B). A deletion variant of CYCLOPS (amino acids 84–366) also interacted with CCaMK, recapitulating previous observations^35^. The control p53 and SV40T Gal4 fusions also interacted, while no interaction was observed in negative controls. These results demonstrate that the modular MK system can be used for co-expression in bacteria and for yeast two-hybrid assays in yeast to assess protein-protein interactions.

### Protein localization in *Trypanosoma brucei*

The MK system was also used for experiments with the protozoan parasite *Trypanosoma brucei*, a pathogen causing fatal tropical diseases in humans and livestock^36^. During its complex, digenetic life cycle the parasite proliferates as ‘long slender bloodstream form’ in the blood of a mammalian host and as ‘procyclic form’ in the digestive tract of a tsetse fly, a blood-sucking insect responsible for trypanosome transmission. A plasmid for tetracycline-inducible expression of the *T. brucei* protein kinase A regulatory subunit (PKAR) fused N-terminal to mNeonGreen (mNG) was assembled for integration into the trypanosome genome (Fig. 6C). The PKAR-mNG fusion localized along the trypanosome flagellum in both life cycle stages (Fig. 6D), in agreement with previous localization^37,38^ and proteome analyses^39^ in procyclic trypanosomes. In-gel fluorescence (Fig. 6E) revealed tightly regulated expression with PKAR-mNG (~80 kDa) only detectable upon tetracycline induction in both life cycle stages at a comparable level.

## Discussion

We demonstrate that by creating six independent plasmid backbone series, a variety of experiments in a range of model species can be conducted with the MK system. Therefore, a standard molecular biology laboratory using the MK system would only require cloning a gene of interest once for diverse assays across kingdoms. As all of the backbones utilize the same overhangs, each LI part can be cloned into any of the LII backbones. Further, transcription units in any LII backbone can be combined with LII assemblies in any other backbone series to generate a LIII plasmid. The MK cloning system is thus versatile and economical since plasmids and parts can be used across the platform. In order to perform experiments in a new organism, the required parts for expression (generally promoters and terminators) are cloned and combined with existing components of the MK system. To demonstrate the flexibility of the MK system, we performed a variety of experiments in organisms from multiple kingdoms. We validated the system by performing genome editing (plants), recombinant protein expression (bacteria), protein interaction assays (yeast), protein localization (human cells and trypanosomes), and electrophysiology (African clawed frog). These examples included episomally-maintained and genome-integrated vectors and a variety of requirements for species-specific promoters and regulatory elements for posttranscriptional control. The results of these assays demonstrate that converting existing modular cloning systems for work in a new organism may only require a new backbone and a small number of parts.

The MK system offers a number of advantages beyond the ability to work across kingdoms. First, it utilizes a single default universal backbone (p641-BpiI), reducing the number of LI backbones as the required overhangs are initially added by PCR. Second, additional alternative LI universal backbones and parts were created to circumvent mutagenesis of sensitive parts in special circumstances. Generally, parts must be cleared of at least BsaI sites to allow high-fidelity assembly of LII plasmids. BpiI sites should also be removed by default, but is only necessary if a LIII plasmid will be constructed. Further, one can generate Esp3I- or BsaI-ready LIII plasmids with the MK system by inserting either a *lacZ* (for Esp3I cloning and blue-white screening) or a *ccdB* cassette (for BsaI cloning and negative selection) in replacement of an A-B or C-D part (Supplementary Fig. S5). If such LII or LIII plasmids are premade with predefined parts, then a LI part only needs to be cleared of one enzyme site (Esp3I or BsaI if present) as a means of minimizing mutagenesis. Third, we have created parts and a backbone compatible with Gateway cloning, which relies upon recombination (no mutagenesis required) and offers the advantage of large collections of destination plasmids. The MK system includes a compatible entry plasmid (pENTR-BsaI-Tet) that supports the simple conversion of LI C-D modules into entry clones. Beyond entry cloning, we also combined the modular capabilities of Golden Gate with Gateway as demonstrated previously^14,20,40^. The A-B or C-D *attR1-ccdB-Tet-attR2* LI parts can be inserted into LII by standard BsaI cloning with other LI parts to create LII or LIII destination plasmids. This strategy allows one to create custom destination plasmids which can then be recombined with existing Gateway entry clone collections.

In conclusion, we have generated a flexible and versatile modular cloning system for use in model species across kingdoms. The MK system simplifies the cloning process, saving time and resources in the laboratory. The MK system relies on the same modular cloning principles as other Golden Gate systems but is applicable to a wider range of model organisms. The increased range of the system allows further use of components across the platform and offers a streamlined workflow for generating complex DNA assemblies.

## Methods

### Plasmid backbone and part preparation

The p641-Bpi and p641-Esp3I universal LI backbones were generated by inserting a *ccdB* negative selection cassette into pSEVA641 (http://wwwuser.cnb.csic.es/~seva/). LII and LIII bacterial plasmids are based upon pSEVA421/221 (RK2 origin), pSEVA431/231 (pBBR1 origin), pSEVA441/241 (ColE1 origin), and pSEVA451/251 (RSF1010 origin)^29^. LII and LIII plasmids were generated by inserting a Golden Gate compatible *ccdB* cassette amplified from the LIIβ or LIIIβ series of plasmids^10^ into the AvrII/SacI sites. The pPlant series was previously generated^10^ and renamed for this manuscript. The pYeast LII series of plasmids were built in two steps using components of a yeast toolkit^23^. First, each backbone plasmid was built using LI parts followed by the insertion of a *ccdB* cassette. New DNA parts were either generated by PCR amplification from existing plasmids or synthesized as gBlocks (Integrated DNA technologies). All backbone plasmids are listed in Supplementary Table 1. Supplementary Table 2 lists all LII and LIII plasmids used in this study.

### HEK cell transfection and imaging

HEK293T cells were cultured in DMEM supplemented with 10% FBS and 1X Pen/Strep (Sigma-Aldrich). For imaging, cells were plated on 8-well glass bottom imaging slides (Ibidi). Cells were transfected using Lipofectamine 3000 (Thermofisher), following the manufacturer's procedure. For 96-well plates, 40 ng of DNA was transfected using 0.1 μl of Lipofectamine and 0.1 μl of P3000 reagent per well, and left to incubate with cells overnight. Images were acquired on a Nikon spinning disk confocal equipped with a 60 × 1.49NA Apo TIRF oil-immersion objective, an iXon 888 EMCCD camera (Andor) and a CSU-W spinning disk head unit (Yokogawa). Cells were imaged using DIC optical settings (in widefield mode), and using the 488- and 561-nm lasers to excite YFP and mCherry respectively (in confocal mode), with standard filter sets. Images were processed in Fiji. Briefly, fluorescence images were background subtracted using a rolling ball function. Then, fluorescence images were given green (for YFP) or red (mCherry) LUTs to generate overlay images with their respective DIC images, and saved as RGB tiffs.

### Functional analysis in *Xenopus laevis* oocytes

Capped cRNA synthesis, oocyte injection and two-electrode voltage-clamp recordings were performed as described^41,42^. cRNA was synthesized with a mMESSAGE mMACHINE T7 Transcription Kit (Thermo Fisher). Oocytes were injected using a General Valve Picospritzer III (Parker Hannifin Corp) with approximately 25 ng cRNA or with RNase-free water (negative control). Injected oocytes were stored at 18 °C in ND96 solution (96 mM NaCl, 2 mM KCl, 1 mM CaCl2, 1 mM MgCl_2_, 5 mM HEPES, pH 7.4/NaOH, adjusted to 220 mOsm/l with sorbitol), supplemented with 25 µg/ml gentamycin. Current measurements were performed 2 to 3 days after injection using a Turbo Tec-10Cx amplifier (NPI Electronic GmbH). During the measurement, oocytes were perfused with bath solution containing 3 mM, 10 mM, 30 mM or 100 mM KCl, 1 mM CaCl_2_, 10 mM MES, pH 6.2/Tris and osmolarity adjusted to 220 mOsm/l with sorbitol. Expression was confirmed by fusing YFP to the C-terminus of KAT1 and imaging of oocytes using confocal microscopy 2 to 3 days after injection (excitation: 488 nm, detection: 525–575 nm).

### Plant transformation

*Agrobacterium tumefaciens* strain AGL1^43^ was electroporated with LII plasmids and grown on LB media supplemented with rifampicin (50 µg/ml), carbenicillin (50 µg/ml), and spectinomycin (100 µg/ml). *A. tumefaciens* was then grown overnight, washed, and resuspended in infiltration buffer (10 mM MgCl_2_, 10 mM MES-KOH pH 5.6, 150 μM acetosyringone). *Nicotiana benthamiana* leaves were infiltrated with a bacterial cell suspension (final OD_600_ of 0.25 for each plasmid) and returned to a growth chamber (22 °C). After two days, leaf material was harvested and genomic DNA extracted using CTAB. The target site within the *NbPDS1* gene was amplified by PCR (Phusion, NEB) using 100 ng gDNA template (primers listed in Supplementary Table 3). The PCR product was purified (GeneJET Gel Extraction Kit, Thermo Fischer Scientific) and the amplicons digested with MlyI (NEB) in a 10 µl reaction (200 ng DNA). The digestion reaction was then separated by gel electrophoresis. MlyI-resistant bands were extracted from the agarose gel, cloned into the pENTR-BsaI^10^ backbone, and sequenced.

### Recombinant protein expression and purification

The LIII plasmid containing StrepII-SUMO-CYCLOPS and His-SUMO-CCaMK was transformed into BL21 Rosetta cells (Merck). Cells were grown at 37 °C to OD_600_ 0.4, induced with 0.5 mM IPTG, and moved to 22 °C for 4 hours. The cultures were then centrifuged and the pellets frozen at −20 °C. The cells were resuspended in lysis buffer (100 mM Tris pH 7.8, 150 mM NaCl, 5 mM DTT, 1 mM EDTA) with EDTA-free protease inhibitor (Roche) and lysed with a French press. The lysate was centrifuged at 40,000 rpm for 30 minutes with a THC-641 rotor at 4 °C. The supernatant was incubated with 5 ml of Strep-Tactin sepharose beads (IBA Lifesciences) for 1 hour with rotation at 4 °C. The slurry was then placed in a gravity column and washed with 10 mL wash of wash buffer (100 mM Tris pH7.8, 150 mM NaCl, 5 mM DTT). Protein was eluted in 4 elution steps with elution buffer (Wash buffer + 2.5 mM desthiobiotin) and pooled. The eluted proteins were dialyzed to reduce salt content, injected into a Heparin column (Hitrap Heparin HP, GE Healthcare) and eluted with a salt gradient (A: 50 mM Tris pH7.8, 1 mM DTT; B: 50 mM Tris pH7.8, 2 M NaCl, 1 mM DTT). Protein pooled fractions were concentrated with an Amicon Ultra 4 filter device (Merck) with a cut-off of 10 kDa and were then resolved on a Superose S6 SEC column (Superose 6 increase 10/300 GL, GE Healthcare) with Buffer S6 (50 mM Tris pH7.8, 150 mM NaCl, 4 mM DTT).

### Yeast two-hybrid assay

Yeast two-hybrid interaction assays were conducted with the haploid yeast strain AH109 (Clontech). LI part plasmids containing yeast promoters, Gal4AD, GAL4BD, and terminators were combined into either pYeast 2 µ Leu 1-2 or pYeast 2 µ Trp 3-4. All plasmids used for the interaction and complementation assays are listed in Supplemental File 2. Plasmids were transformed using the lithium acetate method^44^ and selected on media lacking leucine and tryptophan (-LW). The interacting protein pair of CCaMK and CYCLOPS from *Lotus japonicus* was used for analysis^35^ along with p53 and SV40T controls^45^. Positive transformants were restreaked on -LW, then used to inoculate overnight cultures in liquid -LW media. Overnight cultures were diluted to OD_600_ of 0.5 in sterile water and diluted 10-fold. 5 μl was spotted on –LW or solid media lacking leucine, tryptophan, adenine, and histidine (−LWAH). Yeast plates were incubated at 28 °C for 4 days.

### *Trypanosoma brucei brucei* culture conditions

Bloodstream forms of the *Trypanosoma brucei brucei* strain AnTat 1.1E were cultivated at 37 °C and 5% CO_2_ in modified HMI-9 medium^46^ supplemented with 10% (v/v) heat-inactivated fetal bovine serum (FBS). Cell density was monitored using a haemocytometer and was kept below 8 × 10^5^/ml for continuous growth of the replicative long slender bloodstream form stage. Differentiation to the insect-infective procyclic stage was initiated by density-dependent transformation of long slender bloodstream forms to growth-arrested short stumpy bloodstream forms (culture with starting density of 5 × 10^5^/ml was grown for 36 hours without dilution). Short stumpy forms were transferred into modified DTM medium^47^ complemented with 15% (v/v) heat-inactivated FBS at 2 × 10^6^/ml, followed by addition of 6 mM cis-aconitate and cultivation at 27 °C. The resulting procyclic forms were grown at 27 °C in SDM-79 medium^48^ supplemented with 10% (v/v) heat-inactivated FBS.

### Cloning and generation of transgenic trypanosomes

Two copies of a tetracycline repressor were integrated into the *T. brucei* AnTat 1.1E genome by transfection^49^ with the NotI-linearized plasmid pHD1313^50^. Antibiotic selection was performed with 10 µg/ml phleomycin. *T. brucei* PKAR (GenBank AF182823) was expressed as C-terminal mNeonGreen fusion using a tetracycline-inducible Golden Gate vector that is partially based on the inducible expression vector pHD615^51^. Briefly, six different entry vectors in backbone p641-Bpi were generated that contained: (1) a sequence for homologous recombination and integration into an 18 S ribosomal DNA spacer, a promoter from the PARP surface protein gene family, the *E. coli* tetracycline operator, and 5′UTR sequences from a PARP gene including the *trans*-splicing site (A-B fragment); (2) *T. brucei* PKAR ORF (C-D fragment); (3) mNeonGreen ORF for C-terminal tagging (D-E fragment); (4) VSG117 3′UTR regulatory sequences from the VSG117 gene, PARP poly(A)-addition region from a PARP gene, and a VSG221 promoter (E-F fragment); (5) puromycin resistance gene (PAC) including flanking actin 5′ and 3′UTR sequences (F-G fragment). Fragments 1 and 4 were amplified from pHD615^51^, PKAR ORF from *T. brucei* AnTat 1.1 genomic DNA with removal of an internal BpiI site by site-directed mutagenesis. To generate a Tet-inducible PKAR-mNeonGreen construct, the entry vectors were cloned into the backbone BB30^10^ (Amp^R^ and containing a ColE1 origin). The resulting plasmid was linearized with SphI for transfection and trypanosomes were selected with 0.1 µg/ml puromycin. Expression of the PKAR-mNeonGreen fusion protein was induced by treatment with 1 µg/ml tetracycline for 24 h prior to downstream analysis.

### In-gel fluorescence and microscopy

Trypanosomes were lysed in 1 × Laemmli sample buffer (125 mM Tris pH 6.8, 4% (w/v) SDS, 20% (v/v) glycerol, 10% 2-mercaptoethanol, 0.02% (w/v) bromophenol blue) at a density of 3 × 10^5^/µl. Samples were sonicated (Bioruptor®, Diagenode (Belgium); settings: high energy, 4 cycles, 30 s on/off) and immediately subjected to 10% SDS PAGE. The gel was scanned with a Typhoon Trio Variable Mode Imager System (GE Healthcare) at λ_ex_ = 488 nm and λ_em_ = 526 nm for mNeonGreen and at λ_ex_ = 670 nm and λ_em_ = 633 nm for visualization of the Blue Prestained Protein Standard (NEB). For fluorescence microscopy, *T. brucei* bloodstream forms were fixed in 2% paraformaldehyde, whereas procyclic forms were subjected to live cell imaging in PBS. Cells were imaged using a DeltaVision Elite widefield fluorescence microscope (GE Healthcare) equipped with a CoolSnap HQ2 CCD camera (Photometrics, Arizona, USA) and images were processed with ImageJ^52,53^.

## Supplementary information

Supplementary Information

## Data Availability

The MK system plasmid backbone sequences have been deposited at GenBank, accessions MK495748-MK495798. Plasmids used in this study are available at Addgene (https://www.addgene.org/) for distribution.
