# Supplementary material for: A unified multi-kingdom Golden Gate cloning platform
Source: Sci Rep. 2019 Jul 12;9:10131. doi: 10.1038/s41598-019-46171-2 (PMC6626145; doi:10.1038/s41598-019-46171-2)
Supplement: Supplementary file 1 — Supplementary Information [file 41598_2019_46171_MOESM1_ESM.pdf]

Supplementary Information

A unified multi-kingdom Golden Gate cloning platform

David Chiasson, Victor Giménez-Oya, Martin Bircheneder, Sabine Bachmaier, Tanja Studtrucker, Joel Ryan, Katharina Sollweck, Heinrich Leonhardt, Michael Boshart, Petra Dietrich, Martin Parniske

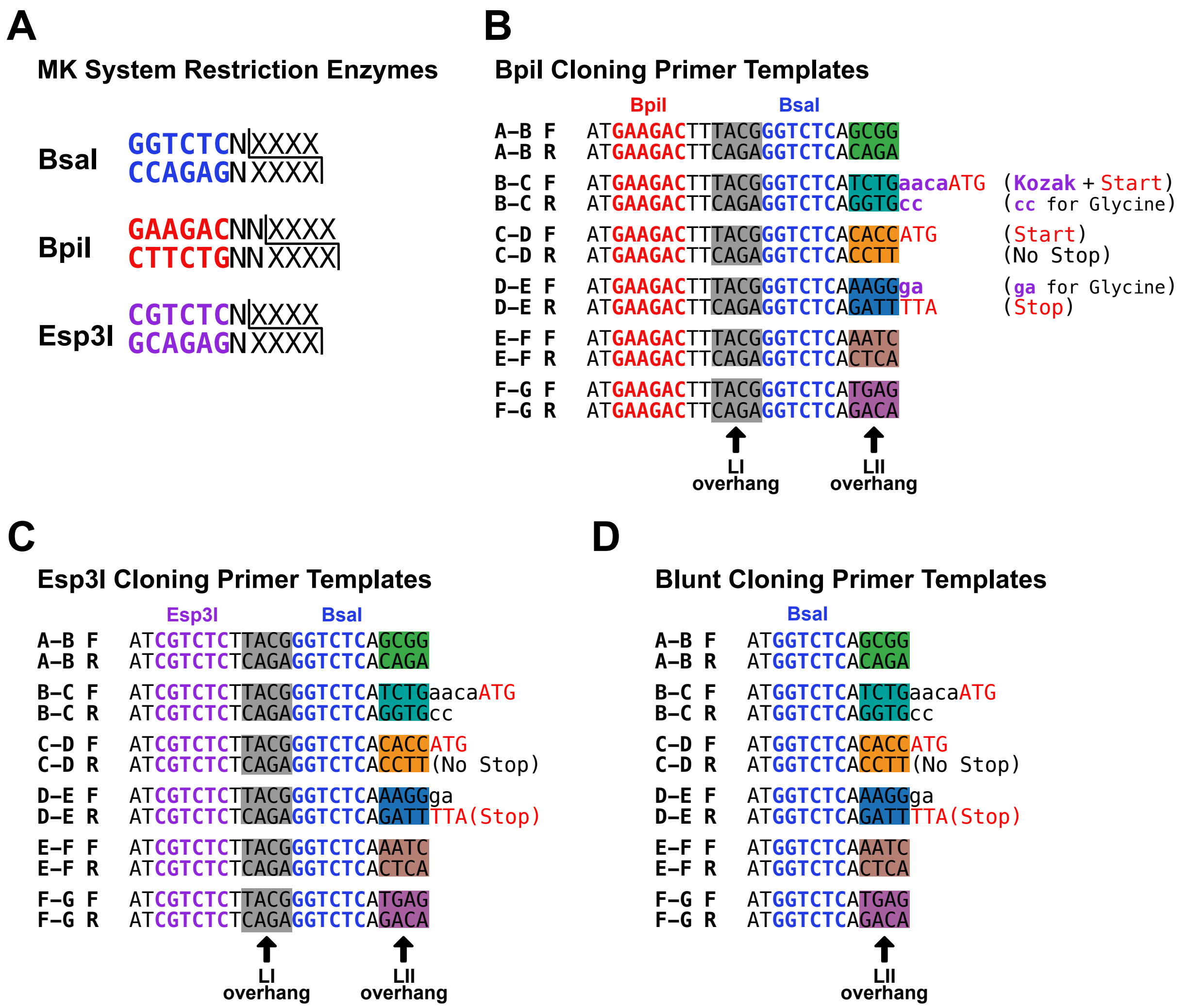

Supplemental Figure 1. Primer design for entry cloning. (**A**) Overview of the enzymes used in the MK system showing their recognition sites and cutting properties. All enzymes cut outside of their recognition site to generate a 4 bp staggered overhang. 'X' in the overhang denotes any nucleotide which can be custom designed. When amplifying a DNA part for LI cloning, primer templates for BpiI (**B**), Esp3I (**C**), and blunt cloning (**D**) are added to the 5' end of each primer. The indicated LI overhang is for sticky, directional cloning, while the LII overhang delineates the part position. A eukaryotic Kozak sequence (AACA) is added 5' to B-C parts. The stop codon for a C-D part is removed and is added 3' of D-E parts. Two nucleotides must be added to the B-C R and D-E F primers to maintain the reading frame when making protein fusions (encode for glycine residues).

A

LI Cloning Workflow

Amplify fragment(s) with proof-reading DNA Polymerase
↓
Column clean-up or gel extract
↓
Perform BpiI cut-ligation in PCR tube
↓
Transform ccdB-susceptible strain
↓
Pick 1-2 colonies, grow overnight
↓
Plasmid prep and restriction digest
↓
Sequence insert

B

LII/LIII Cloning Workflow

Prepare cut-ligation in PCR tube
↓
Perform BsaI, BpiI, or Esp3I cut-ligation
↓
Transform ccdB-susceptible strain
↓
Pick 1-2 colonies, grow overnight
↓
Plasmid prep and restriction digest
↓
Sequence clone

C

**BpiI Cut-Ligation Protocol**		**BsaI Cut-Ligation Protocol**		**Esp3I Cut-Ligvation Protocol**	
Backbone (100ng/µl)	1.0 µl	Backbone (100ng/µl)	1.0 µl	Backbone (100ng/µl)	1.0 µl
Insert(s) (100 ng/µl)	1.0 µl	Insert(s) (100 ng/µl)	1.0 µl	Insert(s) (100 ng/µl)	1.0 µl
10x Buffer G	1.5 µl	10x CutSmart Buffer	1.5 µl	10x Tango Buffer	1.5 µl
10 mM ATP	1.5 µl	10 mM ATP	1.5 µl	10 mM ATP	1.5 µl
BpiI	0.75 µl	BsaI	0.75 µl	Esp3I	0.75 µl
T4 Ligase	0.75 µl	T4 Ligase	0.75 µl	T4 Ligase	0.75 µl
H_2O		H_2O		H_2O	
Total	15.0 µl	Total	15.0 µl	Total	15.0 µl
37°C for 10 min / 16°C for 10 min (x 6-8 cycles)		37°C for 10 min / 16°C for 10 min (x 6-8 cycles)		37°C for 10 min / 16°C for 10 min (x 6-8 cycles)	
37°C for 10 min		37°C for 10 min		37°C for 10 min	
65°C for 20 min		65°C for 20 min		65°C for 20 min	
10°C hold		10°C hold		10°C hold	
Transform 5 µl into *E. coli*		Transform 5 µl into *E. coli*		Transform 5 µl into *E. coli*	

Supplemental Figure 2. Workflow and protocols for cloning. Shown are the standard workflows for (**A**) LI and (**B**) LII/LIII cloning. (**C**) Protocols for performing cut-ligations with BpiI (Thermo Fisher Scientific), BsaI-HF (New England Biolabs), and Esp3I (Thermo Fisher Scientific) enzymes. By default, all backbone and entry plasmids are diluted to 100 ng/µl and 100 ng of each is used per cut-ligation. If the efficiency is low, the backbone can be kept at 100 ng while adding a molar ratio of 2:1 (part:backbone) of each insert. Cut-ligations are performed in a thermocycler for 6-8 cycles.

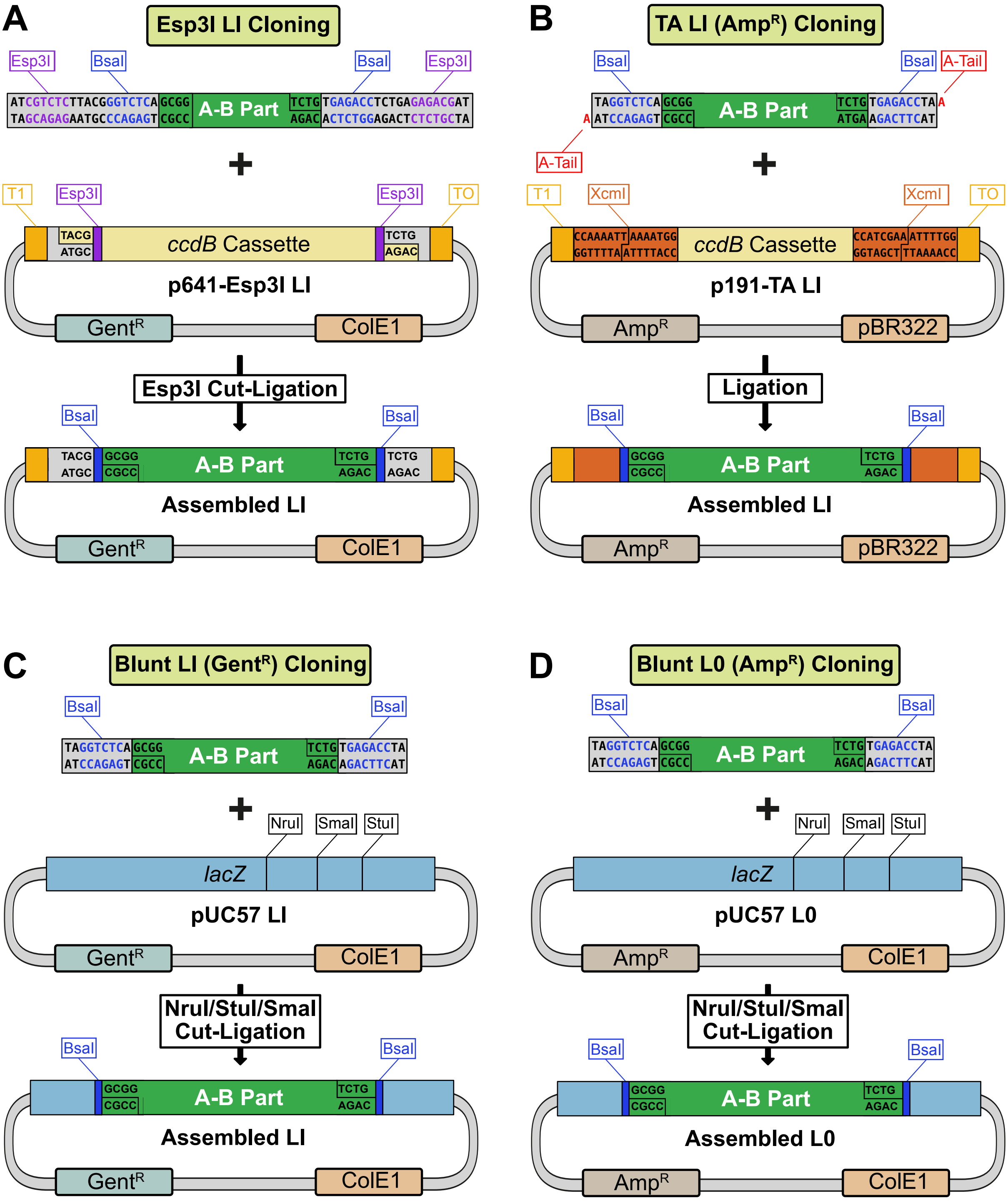

Supplemental Figure 3. Alternative LI entry cloning strategies. (**A**) If a part contains numerous BpiI sites and no (or few) Esp3I sites, it can be cloned into p641-Esp3I by Esp3I cut-ligation. To avoid performing mutagenesis, TA (**B**) or blunt cloning (**C, D**) can be utilized. (**B**) The p191-TA LI backbone is prepared by digesting with XcmI to generate 5' T overhangs and gel purified. A 3' A nucleotide can be added to blunt PCR products by Taq Polymerase in the presence of dATP. A standard ligation is then performed. Blunt cloning (NruI, SmaI, or StuI enzymes) in combination with blue-white screening can be performed with a modified pUC57 backbone which is either gentamycin (**C**) or ampicillin (**D**) resistant.

LIII Backbones

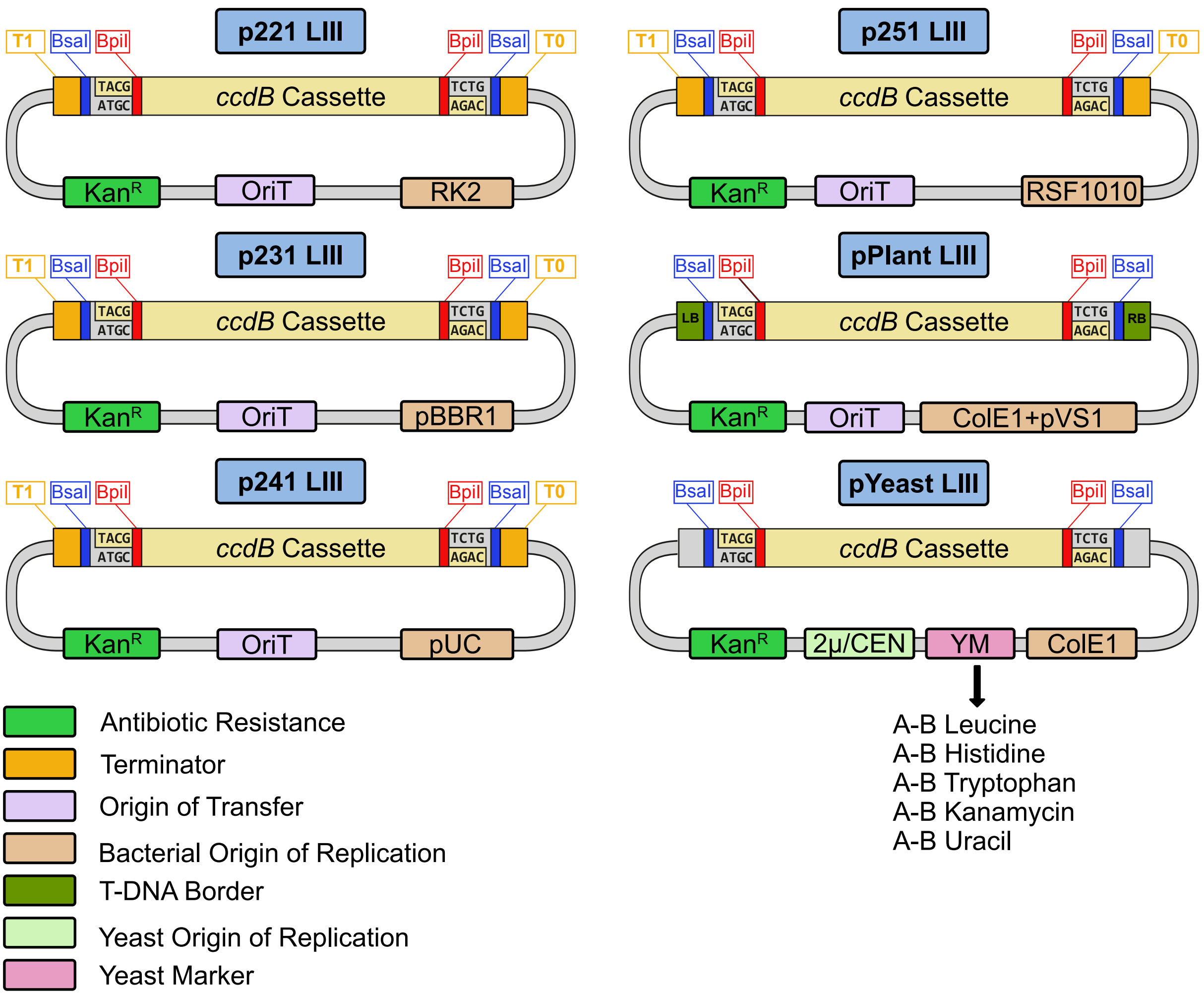

Supplemental Figure 4. MK system backbones for LIII cloning. LIII (A-B position) plasmid backbones are identical to LII backbones except for the kanamycin resistance cassette and reversed orientation of the BsaI and BpiI sites. Bacterial plasmids contain an origin of transfer (OriT) and flanking terminators (T1, T0). Plant backbones contain a double origin of replication for *E. coli* and *Agrobacterium* along with an OriT and T-DNA borders for genomic integration. Yeast backbones contain an additional yeast origin of replication (high copy 2µ or low copy CEN/ARS) along with an auxotrophic or dominant marker.

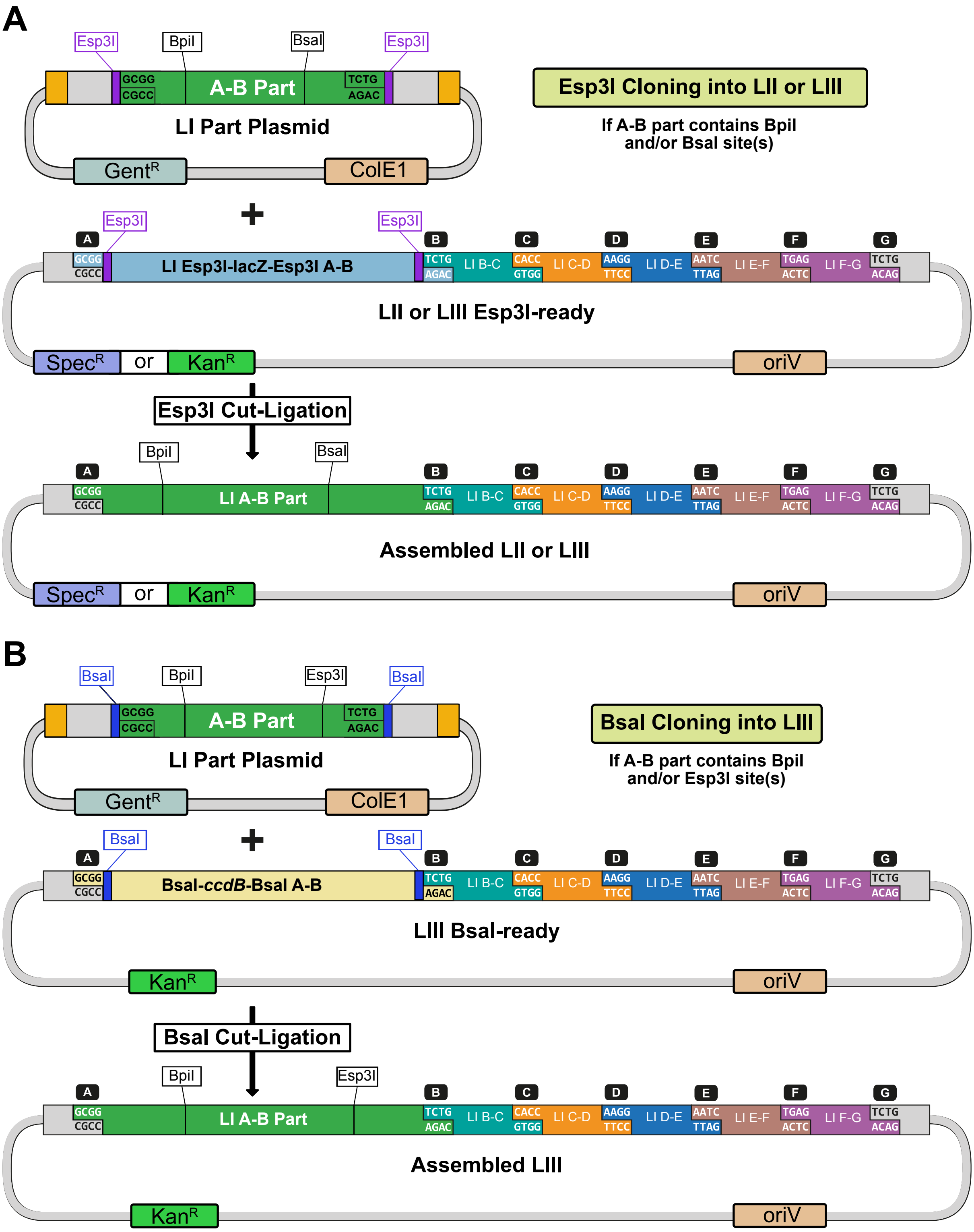

Supplemental Figure 5. Alternative cloning strategies for parts with sensitive BsaI, BpiI, or Esp3I sites. (**A**) For bypassing BsaI or BpiI cut-ligations with sensitive A-B parts containing BsaI or BpiI sites, the LI Esp3I-lacZ-Esp3I A-B DMY is cloned into a LII in place of an A-B promoter (C-D lacZ DMY also available). The A-B part is cloned with flanking Esp3I sites, and sub-cloned into either the LII or LIII plasmid containing the A-B lacZ DMY by Esp3I cut-ligation (blue-white selection). (**B**) If the A-B part contains sensitive BpiI or Esp3I restriction sites, a BsaI-*ccdB*-BsaI A-B DMY is can be inserted by Esp3I cut-ligation to replace the Esp3I-lacZ-Esp3I A-B DMY. The A-B promoter part can then be sub-cloned from LI directly to LIII by BsaI cut-ligation (*ccdB* negative selection).

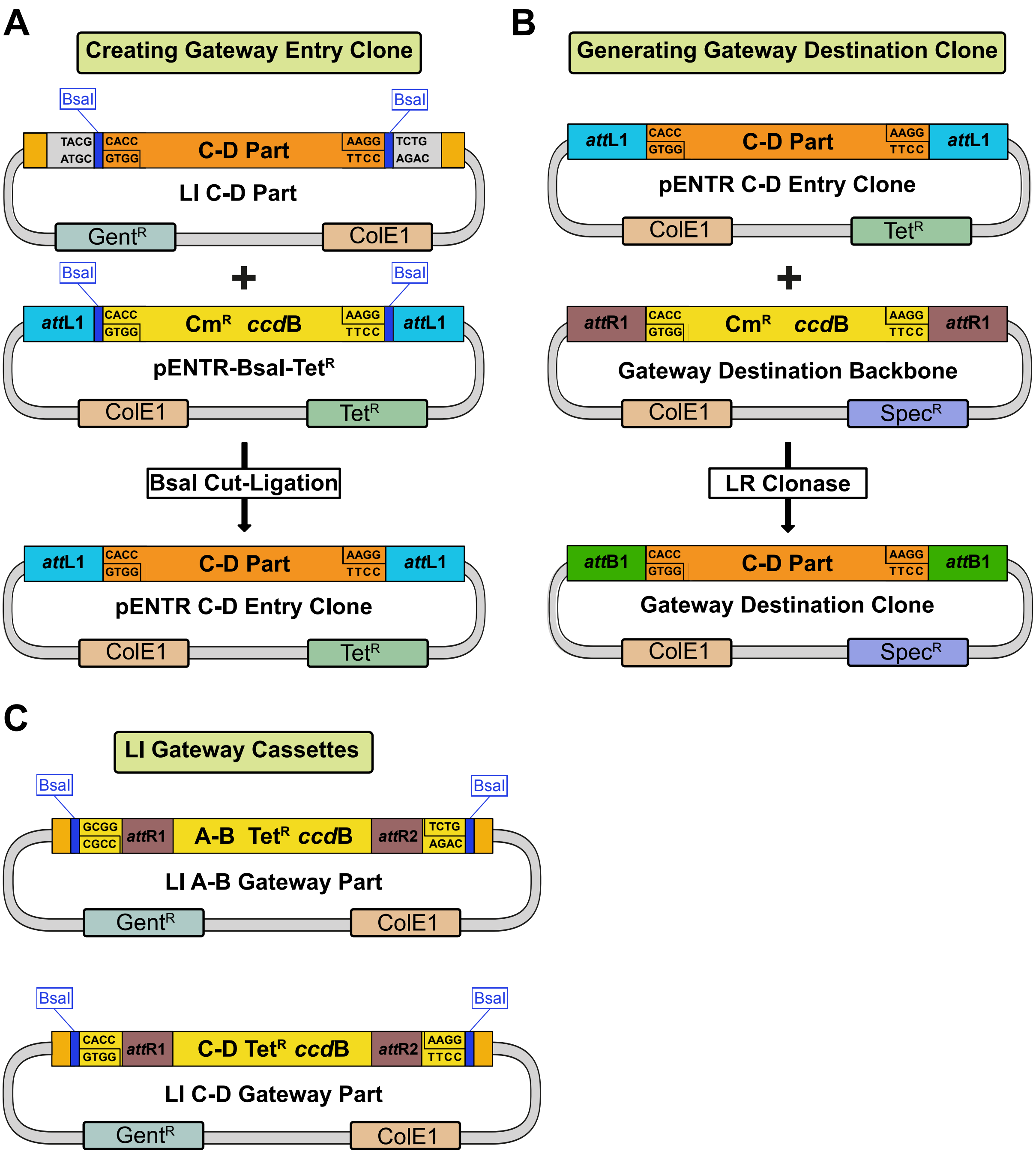

Supplemental Figure 6. Creating Gateway cloning entry and destination plasmids. (**A**) Gateway entry clones can be generated by sub-cloning LI C-D parts (or directly ligating a PCR product) into pENTR-Bsa-Tet. (**B**) A pENTR C-D entry clone is then mixed with a standard Gateway destination backbone and an LR clonase enzyme mix to generate a Gateway destination clone. (**C**) Custom Gateway destination backbones can also be created by Golden Gate cloning. LI A-B or C-D Gateway parts with flanking attR sites are available for cloning into a LII backbone by BsaI cut-ligation.

A

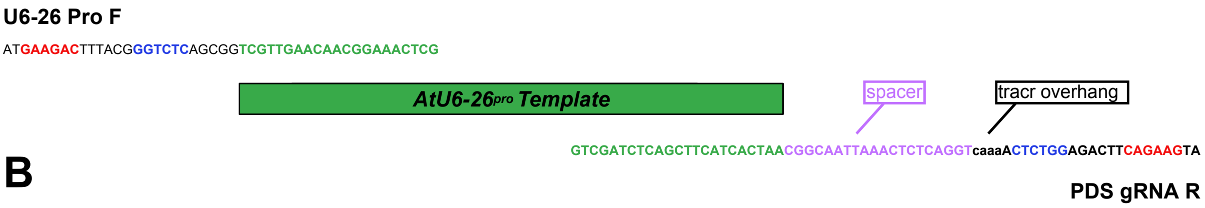

B

**Primer Template (BpiI + BsaI)**	**Target Sequence (PDS)**	**U6 Promoter Template**
AAT**GAAGAC**TTCAGA**GGTCTC**Aaaac	**GCCGTTAATTTGAGAGTCCA**	**aatcactacttcgactctagctg**

Design Reverse Spacer Primer

Step 1. Copy primer template
Step 2. Reverse complement **Target Sequence (PDS)** and add to primer template
Step 3. Add **U6-26 promoter template** sequence

1. AT**GAAGAC**TTCAGA**GGTCTC**Aaaac
2. AT**GAAGAC**TTCAGA**GGTCTC**Aaaac**TGGACTCTCAAATTAACGGC**
3. AT**GAAGAC**TTCAGA**GGTCTC**Aaaac**TGGACTCTCAAATTAACGGCaatcactacttcgactctagctg**

Step 4. Amplify AtU6-26 template, clone into LI
Step 5. Combine into LII with tracr-U6-1term + F-G DMY

C

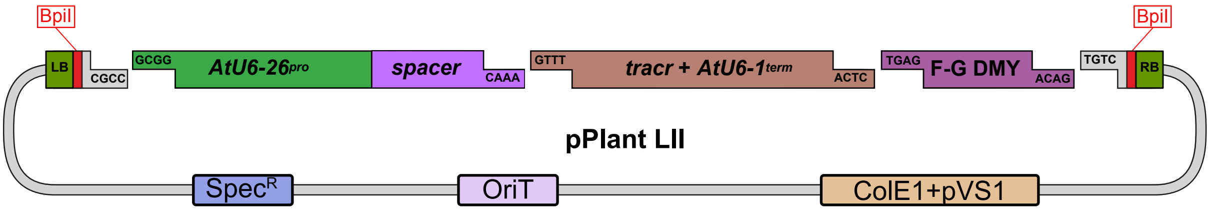

Supplemental Figure 7. CRISPR spacer cloning strategy. (**A**) Overview of the strategy to generate a fusion of the *AtU6-26* (example) promoter and a spacer (target) sequence. The *AtU6-26* promoter is used as a template for PCR in order to add the required flanking sequences. (**B**) Reverse primer design steps to generate a primer that binds to the *AtU6-26* promoter and adds the required spacer sequence. (**C**) The LI *AtU6-26* promoter-spacer fusion is combined with a LI tracr-U6-1 terminator and an F-G DMY in a BsaI cut-ligation to generate a LII plasmid.

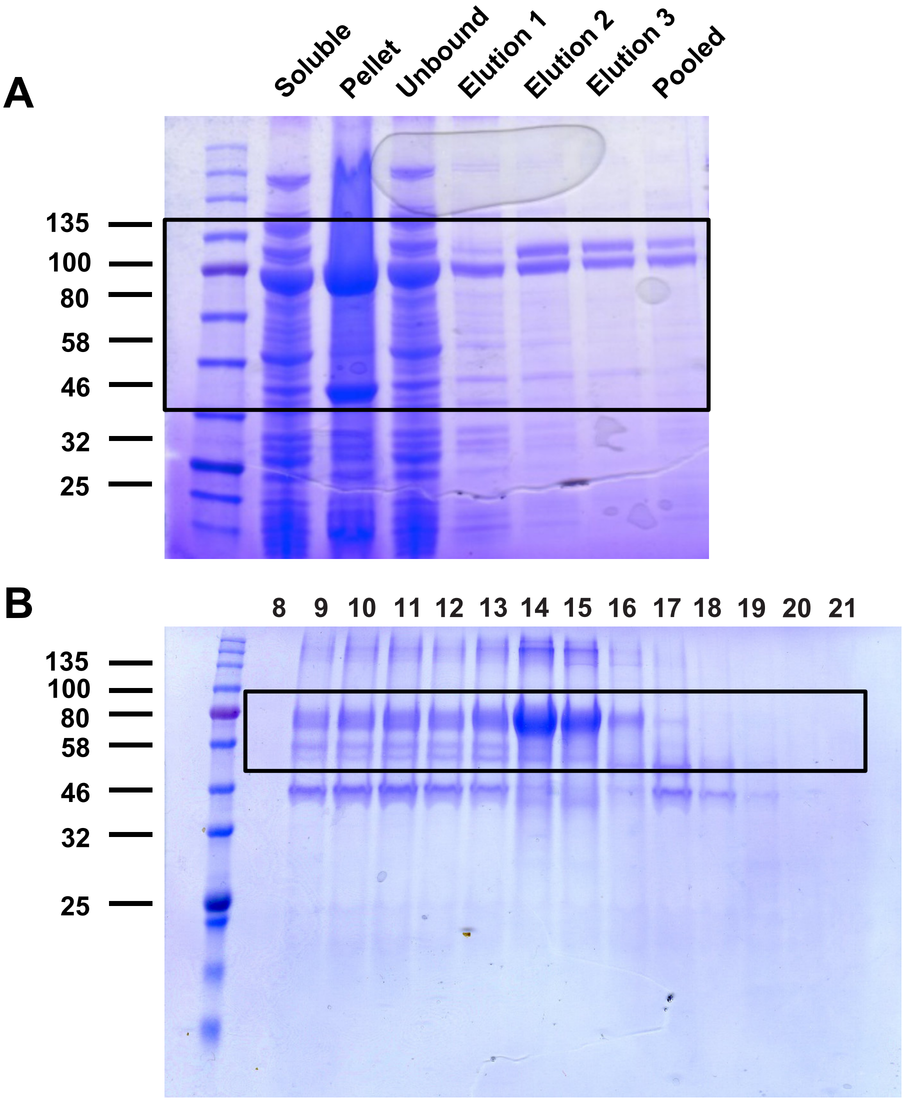

Supplemental Figure 8. Original SDS-PGAE gel images. A. SDS-PAGE separation of StrepII-SUMO-CYCLOPS and His-SUMO-CCaMK proteins after co-expression and purification by StrepII affinity chromatography. **B**. SDS-PAGE separation of StrepII-SUMO-CYCLOPS and His-SUMO-CCaMK protein complex after size exclusion gel chromatography. Molecular weight standards are shown on the left side of both gel images. Black boxes indicate cropped regions chosen for Figure 5.

Supplementary Table 1. Golden Gate backbones used in this study

Backbone	Level	Origin of Replication	Bacterial Resistance	Based Upon	Source
p641-BpiI	I	ColE1	Gentamicin	pSEVA641	SEVA Collection[29]
p641-Esp3I	I	ColE1	Gentamicin	pSEVA641	SEVA Collection[29]
p191-TA	I	pMB1/Rop	Ampicillin	pSEVA191	SEVA Collection[29]
pUC57 LI	I	pUC	Gentamicin	pUC57	Binder et al[10]
pUC57 L0	0	pUC	Ampicillin	pUC57	Binder et al[10]
pENTR-BsaI-Tet	Gateway	ColE1	Tetracycline	pENTR/D-TOPO	Binder et al[10]
p421 F1-2	II	RK2	Spectinomycin	pSEVA421	SEVA Collection[29]
p421 F2-3	II	RK2	Spectinomycin	pSEVA421	SEVA Collection[29]
p421 F3-4	II	RK2	Spectinomycin	pSEVA421	SEVA Collection[29]
p421 F4-5	II	RK2	Spectinomycin	pSEVA421	SEVA Collection[29]
p421 F5-6	II	RK2	Spectinomycin	pSEVA421	SEVA Collection[29]
p221 A-B	III	RK2	Kanamycin	pSEVA221	SEVA Collection[29]
p431 F1-2	II	pBBR1	Spectinomycin	pSEVA431	SEVA Collection[29]
p431 F2-3	II	pBBR1	Spectinomycin	pSEVA431	SEVA Collection[29]
p431 F3-4	II	pBBR1	Spectinomycin	pSEVA431	SEVA Collection[29]
p431 F4-5	II	pBBR1	Spectinomycin	pSEVA431	SEVA Collection[29]
p431 F5-6	II	pBBR1	Spectinomycin	pSEVA431	SEVA Collection[29]
p231 A-B	III	pBBR1	Kanamycin	pSEVA231	SEVA Collection[29]
p441 F1-2	II	ColE1	Spectinomycin	pSEVA441	SEVA Collection[29]
p441 F2-3	II	ColE1	Spectinomycin	pSEVA441	SEVA Collection[29]
p441 F3-4	II	ColE1	Spectinomycin	pSEVA441	SEVA Collection[29]
p441 F4-5	II	ColE1	Spectinomycin	pSEVA441	SEVA Collection[29]
p441 F5-6	II	ColE1	Spectinomycin	pSEVA441	SEVA Collection[29]
p241 A-B	III	ColE1	Kanamycin	pSEVA241	SEVA Collection[29]
p451 F1-2	II	RSF1010	Spectinomycin	pSEVA451	SEVA Collection[29]
p451 F2-3	II	RSF1010	Spectinomycin	pSEVA451	SEVA Collection[29]
p451 F3-4	II	RSF1010	Spectinomycin	pSEVA451	SEVA Collection[29]
p451 F4-5	II	RSF1010	Spectinomycin	pSEVA451	SEVA Collection[29]
p451 F5-6	II	RSF1010	Spectinomycin	pSEVA451	SEVA Collection[29]
p251 A-B	III	RSF1010	Kanamycin	pSEVA251	SEVA Collection[29]
pPlant F1-2	II	ColE1 + pVS1	Spectinomycin	LIIβ F1-2	Binder et al[10]
pPlant F2-3	II	ColE1 + pVS1	Spectinomycin	LIIβ F2-3	Binder et al[10]
pPlant F3-4	II	ColE1 + pVS1	Spectinomycin	LIIβ F3-4	Binder et al[10]
pPlant F4-5	II	ColE1 + pVS1	Spectinomycin	LIIβ F4-5	Binder et al[10]
pPlant F5-6	II	ColE1 + pVS1	Spectinomycin	LIIβ F5-6	Binder et al[10]
pPlant A-B	III	ColE1 + pVS1	Kanamycin	LIIIβ A-B	Binder et al[10]

Backbone	Level	Origin of Replication	Yeast Marker	Bacterial Resistance	Based Upon	Source
pYeast 2µ F1-2	II	ColE1	Leucine	Spectinomycin	Custom Built	Lee et al[23]
pYeast 2µ F2-3	II	ColE1	Histidine	Spectinomycin	Custom Built	Lee et al[23]
pYeast 2µ F3-4	II	ColE1	Tryptophan	Spectinomycin	Custom Built	Lee et al[23]
pYeast 2µ F4-5	II	ColE1	Kanamycin	Spectinomycin	Custom Built	Lee et al[23]
pYeast 2µ F5-6	II	ColE1	Uracil	Spectinomycin	Custom Built	Lee et al[23]
pYeast CEN F1-2	II	ColE1	Leucine	Spectinomycin	Custom Built	Lee et al[23]
pYeast CEN F2-3	II	ColE1	Histidine	Spectinomycin	Custom Built	Lee et al[23]
pYeast CEN F3-4	II	ColE1	Tryptophan	Spectinomycin	Custom Built	Lee et al[23]
pYeast CEN F4-5	II	ColE1	Kanamycin	Spectinomycin	Custom Built	Lee et al[23]
pYeast CEN F5-6	II	ColE1	Uracil	Spectinomycin	Custom Built	Lee et al[23]
pYeast LEU A-B	III	ColE1	Leucine	Kanamycin	Custom Built	Lee et al[23]
pYeast HIS A-B	III	ColE1	Histidine	Kanamycin	Custom Built	Lee et al[23]
pYeast TRP A-B	III	ColE1	Tryptophan	Kanamycin	Custom Built	Lee et al[23]
pYeast KAN A-B	III	ColE1	Kanamycin	Kanamycin	Custom Built	Lee et al[23]
pYeast URA A-B	III	ColE1	Uracil	Kanamycin	Custom Built	Lee et al[23]

Supplementary Table 2. Golden Gate LII and LIII plasmids used in this study

	**AB**	**BC**	**CD**	**DE**	**EF**	**FG**	**Backbone**	**Level**	**Resistance**	**Reference**
GGP1	ELFalpha Pro	mCherry G032	BB7 C-D DMY	BB8 D-E DMY	SV40 Term	SV40:Neomycin	p441 F1-2	II	Spec	Figure 4
GGP2	ELFalpha Pro	NLS G61	YFP	BB8 D-E DMY	SV40 Term	SV40:Neomycin	p441 F1-2	II	Spec	Figure 4
GGP3	T7 β -globin 5'	BB06 B-C DMY	KAT1	YFP G12	β -globin 3'UTR AAA+CCC	BB09 F-G DMY	p441 F1-2	II	Spec	Figure 4
GGP4	T7 β -globin 5'	BB06 B-C DMY	KAT1	YFP G12	β -globin 3'UTR AAA	BB09 F-G DMY	p441 F1-2	II	Spec	Figure 4
GGP5	T7 β -globin 5'	BB06 B-C DMY	KAT1	YFP G12	β -globin 3'UTR	BB09 F-G DMY	p441 F1-2	II	Spec	Figure 4
GGP6	35S Pro A-C		AteCas9 WT	BB8-DE DMY	G45 HSP T	BB09 F-G DMY	pPlant F2-3	II	Spec	Figure 5
	**A-Custom**	**Custom-F**				**FG**	**Backbone**		**Resistance**	
GGP7	AtU6-26 Pro:NbPDS1 gRNA	tracr:U6-1 term				BB09 F-G DMY	pPlant F5-6	II	Spec	Figure 5
GGP8	**AB**	**BC**	**CD**	**DE**	**EF**	**FG**	**Backbone**	**Level**	**Resistance**	
GGP9	T7 lacO	HIS-SUMO	CCaMK	BB08 DMY	T7 Terminator	BB09 F-G DMY	pSEVA431 1-2	II	Spec	Figure 5
GGP10	T7 lacO	StrepII-SUMO	CYCLOPS	BB08 DMY	T7 Terminator	LacI	pSEVA431 3-4	II	Spec	Figure 5
	**1-2**	**2-3**	**3-4**	**4-5**	**5-6**		**Backbone**	**Level**	**Resistance**	
GGP11	GGP9	BB39 2-3 DMY	GGP10	BB40 4-5 DMY	BB65 5-6 DMY		p231 A-B	III	Kan	Figure 5
	**AB**	**BC**	**CD**	**DE**	**EF**	**FG**	**Backbone**	**Level**	**Resistance**	
GGP12	ADH Pro 400	GAL4AD GGS B-C	CYCLOPS	BB08 DMY	TDH1 Term	BB09 FG DMY	pYeast LII 2μ LEU 1-2	II	Spec	Figure 6
GGP13	ADH Pro 400	GAL4AD GGS B-C	CYCLOPS (84-366)	BB08 DMY	TDH1 Term	BB09 FG DMY	pYeast LII 2μ LEU 1-2	II	Spec	Figure 6
GGP14	ADH Pro Truncated	GAL4BD GGS B-C	CCaMK C-D	BB08 DMY	ADH1 Term	BB09 FG DMY	pYeast LII 2μ TRP 3-4	II	Spec	Figure 6
GGP15	ADH Pro Truncated	GAL4BD GGS B-C	p53 (72-390)	BB08 DMY	ADH1 Term	BB09 FG DMY	pYeast LII 2μ TRP 3-4	II	Spec	Figure 6
GGP16	ADH Pro Full length	GAL4AD GGS B-C	SV40T (87-708)	BB08 DMY	ADH1 Term	BB09 FG DMY	pYeast LII 2μ LEU 1-2	II	Spec	Figure 6
GGP17	5'Homology PARP Pro	BB06 B-C DMY	PKAR	mNeonGreen	VSG poly A (PARP)	Puromycin + 3'Homology	pLII-Amp F1-2 (BB30)	II	Amp	Figure 6

Supplementary Table 3. Primers used in this study for genome editing

Name	Sequence 5'-3'	Purpose
U6-26 Pro F	ATGAAGACTTTACGGGTCTCAGCGGTCGTTGAACAACGGAAACTCG	Cloning U6-26 Pro:PDS Spacer
PDS Spacer R	ATGAAGACTTCAGAGGTCTCAaaacTGGACTCTCAAATTAACGGCaatcactacttcgactctagctg	Cloning U6-26 Pro:PDS Spacer
MlyI BsaI F	ATGGTCTCACCTTACATAACAAATTCCTTTGCAAGC	Cloning PDS target region into pENTR-Bsa
MlyI BsaI R	ATGGTCTCACCTTTTTAAAGGATTAAAGTCCTTTGTCA	Cloning PDS target region into pENTR-Bsa
